# The Summer Outing

**Published:** 1890-09

**Authors:** 


### HALL'S JOURNAL OF HEALTH

TRUTH DEMANDS NO SACRIFICE; ERROR CAN MAKE NONE.

Vol. 37. SEPTEMBER, 1890. No. 9.

### THE SUMMER OUTING.

The necessity for recreation and diversion unmixed with the work-a-day avocation of the industrial classes, as well brain workers as craftsmen, has come to be an acknowledged fact, and no country in the world affords greater facilities for their attainment than ours.

We have a great variety of seaboard resorts accessible to the whole country, besides inland watering places, including mineral springs variously infused with special healing properties, mountain and highland regions and lake and river centres of pastime and pleasure, almost without number. Canoeing and bicycling for our young men have attained great popularity, and almost every water-course and highway is alive with happy voyagers, whose white tents dot the shores of many an adjacent refuge.

For such as habitually dwell near the sea, the highlands are found to be better suited to restore their lost vitality, and by the same law, our inland dwellers derive the greater benefit in the change which a season near the salt water brings to them.

The need of intervals of relaxation from continuous labor, is so generally felt and conceded, that it has become almost a matter of course, during some portion of the summer months. The brief intervals which some brain workers allow themselves, do them but little good, for their intellectual faculties have no real rest. It is important that they should get wholly away from their accustomed labors, in thought, in purpose and in action. They should "sink the shop" as the old play has it, and if too aged or too dignified to indulge in the popular out door sports, there are woods, and fields and waters upon every hand, which hold out their inducements for a drive, a ramble or a row. These are too essentially important for the restoration of impaired energies to be neglected. Exercise with diversion is what is most beneficial. Man, on his physical side is a complicated machine whose parts are delicately organized and mutually dependent. Nothing is more destructive to its proper performance than either mental or physical overwork. The usual periods of rest should never be neglected.

The cessation from all worldly pursuits on the seventh day grew out of a physical necessity. It was found that the laborer is unable to recover during the night the oxygen he has expended in the preceding day's work, and that after six days of continual labor, it requires a whole day's rest to re-establish it, and restore his impaired vitality.

One is careful enough to wind his watch, keep its delicate machinery lubricated, and guard it against any severe shock. Should he be less careful of his own organism, which is far more complicated in its parts and delicate in its construction?

